# Supplementary material for: Ubiquitinated-PCNA protects replication forks from DNA2-mediated degradation by regulating Okazaki fragment maturation and chromatin assembly
Source: Nat Commun. 2020 May 1;11:2147. doi: 10.1038/s41467-020-16096-w (PMC7195461; doi:10.1038/s41467-020-16096-w)
Supplement: Supplementary file 1 — Supplementary Information [file 41467_2020_16096_MOESM1_ESM.pdf]

Supplementary Information

Ubiquitinated-PCNA protects replication forks from DNA2-mediated degradation by regulating Okazaki fragment maturation and chromatin assembly

Thakar et al.

Supplementary Figure 1

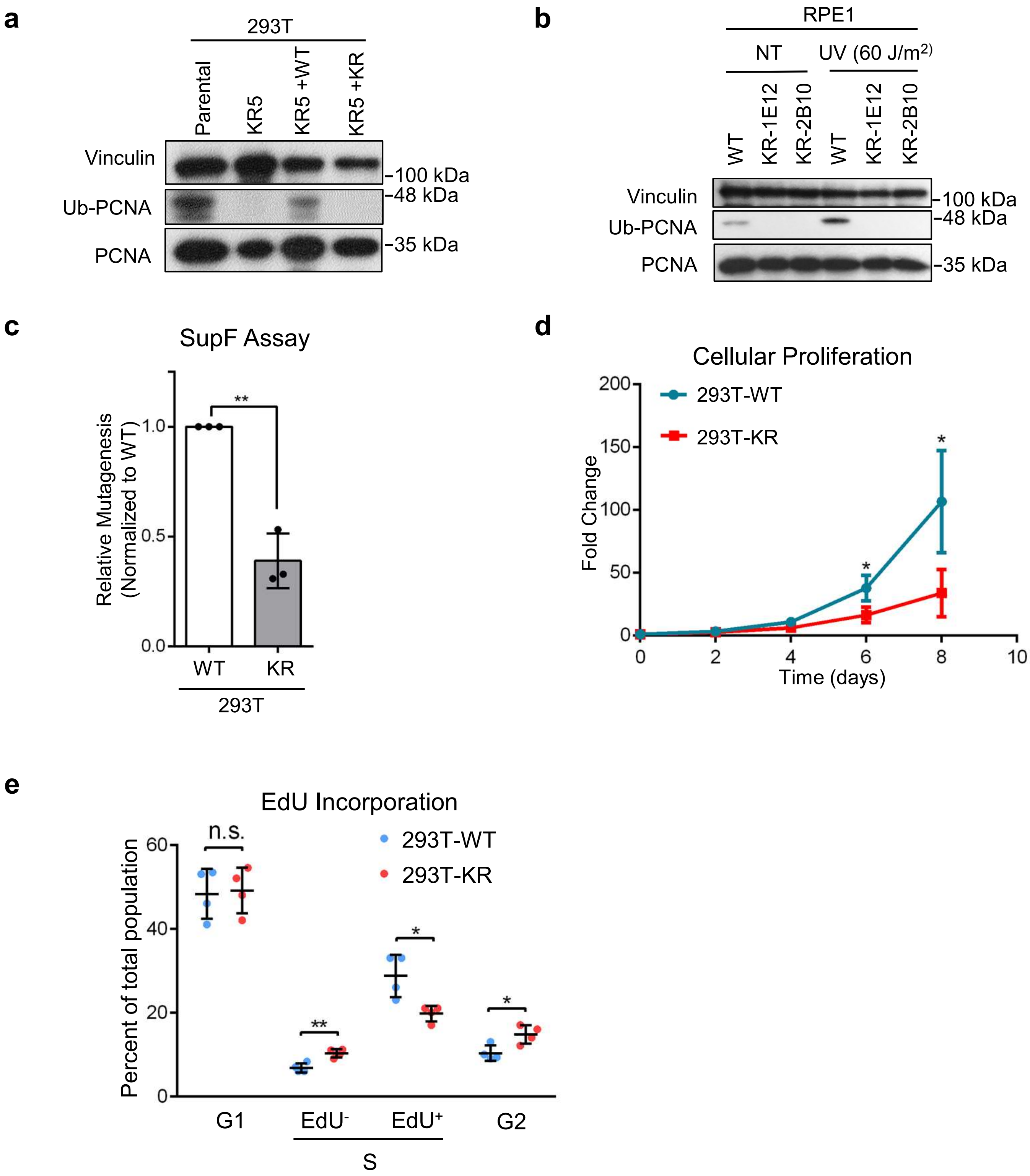

Supplementary Fig. 1. Functional characterization of human PCNA-K164R mutant cells. a. Western blot showing PCNA and ubiquitinated PCNA levels in parental and the KR5 clone obtained through CRISPR/Cas9-mediated editing of the PCNA gene in 293T cells. The KR5 clone was complemented by re-expression of wildtype or K164R PCNA from a lentiviral construct, to restore normal PCNA levels. **b**. Western blot showing the loss of PCNA ubiquitination in two different RPE1-K164R clones. Denatured extracts of cells grown under normal growth conditions, or 3h after exposure to the indicated UV dose, were analyzed. **c**. SupF shuttle plasmid mutagenesis assay showing reduced UV-induced mutagenesis in 293T-K164R cells. The average of three experiments, with standard deviations indicated as error bars, is shown. Asterisks indicate statistical significance (t-test, two-tailed, unequal variance). **d**. Cellular proliferation experiment showing reduced growth rate of 293T-K164R cells under normal growth conditions. The average of three experiments, with standard deviations indicated as error bars, is shown. Asterisks indicate statistical significance (t-test, two-tailed, unequal variance). **e**. EdU incorporation assay showing reduced proportion of cells actively undergoing DNA synthesis under normal growth conditions. The average of four experiments, with standard deviations indicated as error bars, is shown. Asterisks indicate statistical significance (t-test, two-tailed, unequal variance). Source data are provided as a Source Data file.

Supplementary Figure 2

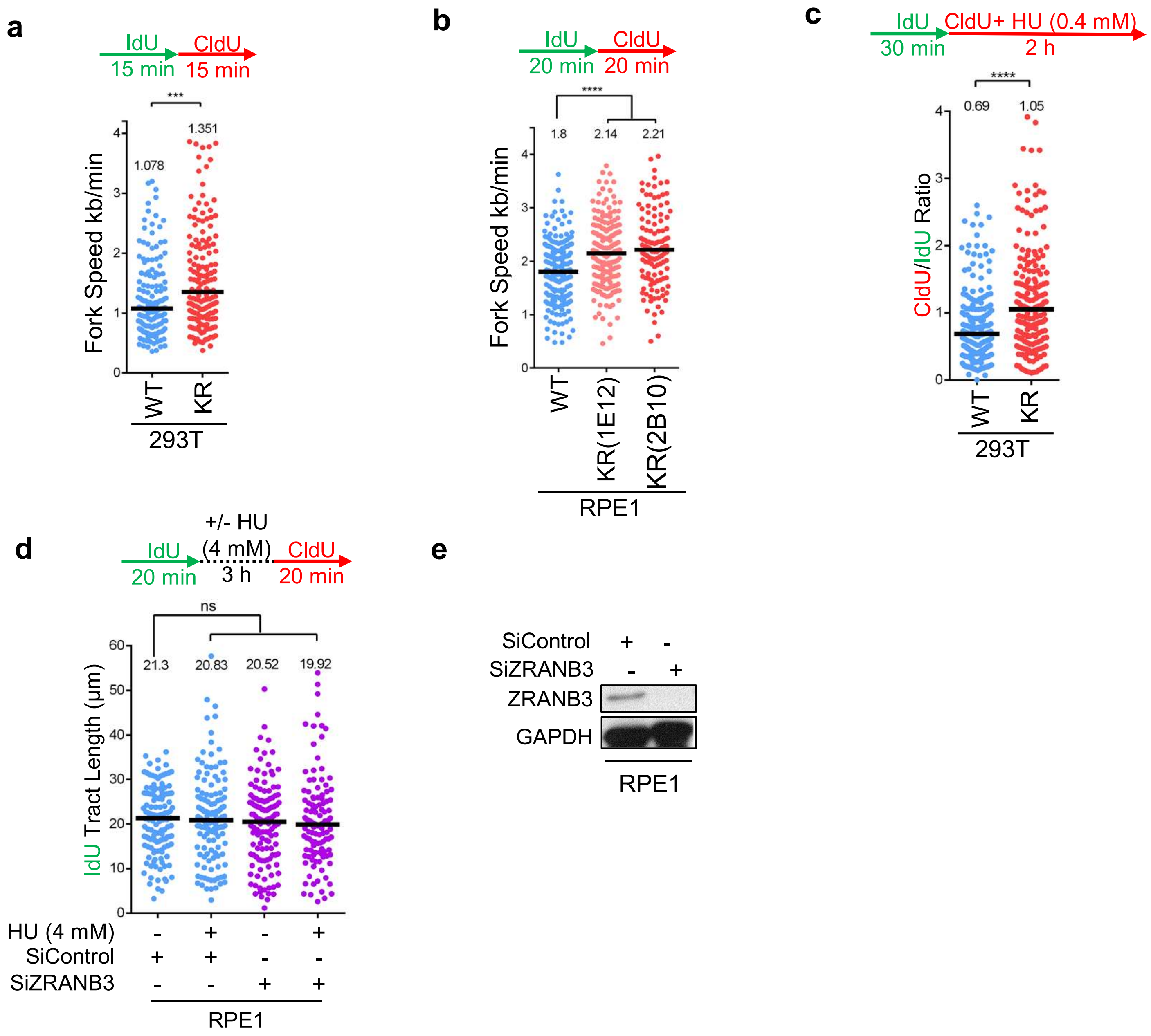

Supplementary Fig. 2. Loss of PCNA ubiquitination results in faster replication forks. a, b. DNA fiber combing experiments showing increased fork speed in 293T-K164R (**a**) and RPE1-K164R (**b**) cells. Each 1µm in fiber length was set to correspond to 2 kilobases of DNA. Median values are marked on the graph and listed at the top. Asterisks indicate statistical significance (Mann-Whitney test, two-sided). Schematic representations of the assay conditions are also presented. **c**. DNA fiber combing experiment showing reduced fork slowing in 293T-K164R cells upon low-level replication stress exposure. The ratio of CldU to IdU tract lengths is presented, with the median values marked on the graph and listed at the top. Asterisks indicate statistical significance (Mann-Whitney test, two-sided). A schematic representation of the assay conditions is also presented. **d**. ZRANB3 depletion does not affect fork speed under normal growth conditions, or nascent strand degradation upon fork arrest. The quantification of the IdU tract length is shown, with the median values marked on the graph and listed at the top. Statistical significance (Mann-Whitney test, two-sided) and a schematic representation of the DNA fiber combing assay conditions are also presented. **e**. Western blot showing ZRANB3 depletion upon siRNA-mediated knockdown.

Supplementary Figure 3

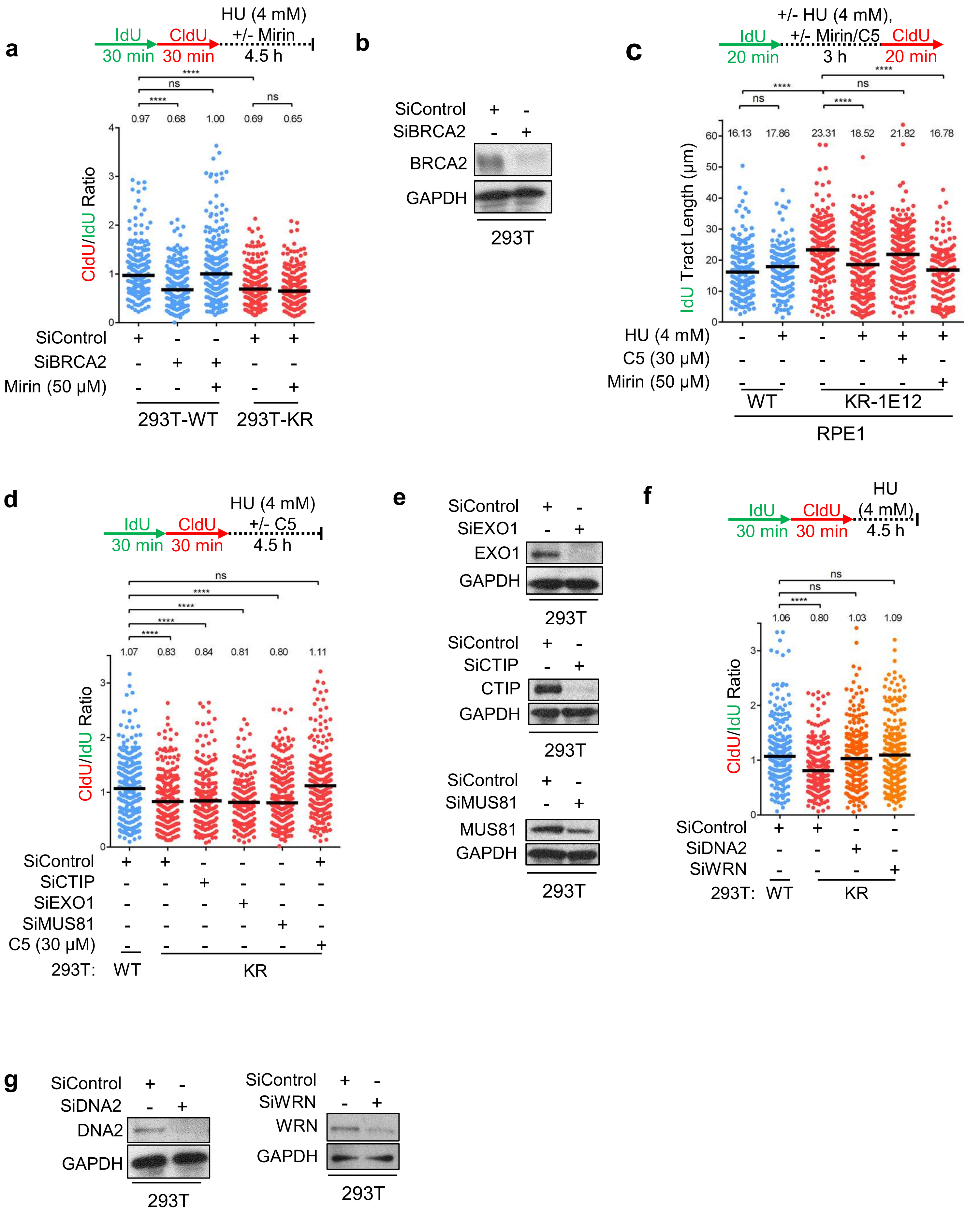

Supplementary Fig. 3. DNA2 degrades stalled replication forks in PCNA-K164R cells. a. MRE11 inhibition by mirin suppresses HU-induced nascent strand degradation in BRCA2-knockdown cells, but not in 293T-K164R cells. The ratio of CldU to IdU tract lengths is presented, with the median values marked on the graph and listed at the top. Statistical significance (Mann-Whitney test, two-sided) and a schematic representation of the DNA fiber combing assay conditions are also presented. **b**. Western blot showing BRCA2 depletion upon siRNA-mediated knockdown. **c**. DNA2 inhibition by C5, but not MRE11 inhibition by mirin, suppresses HU-induced nascent strand degradation in RPE1-K164R cells. The quantification of the IdU tract length is shown, with the median values marked on the graph and listed at the top. Statistical significance (Mann-Whitney test, two-sided) and a schematic representation of the DNA fiber combing assay conditions are also presented. **d**. Depletion of nucleases CTIP, EXO1, and MUS81 does not suppress HU-induced nascent strand degradation in 293T-K164R cells. The ratio of CldU to IdU tract lengths is presented, with the median values marked on the graph and listed at the top. Statistical significance (Mann-Whitney test, two-sided) and a schematic representation of the DNA fiber combing assay conditions are also presented. **e**. Western blots showing CTIP, EXO1, and MUS81 depletion upon siRNA-mediated knockdown. **f.** Knockdown of DNA2 or WRN suppresses the HU-induced nascent strand degradation in 293T-K164R cells. The ratio of CldU to IdU tract lengths is presented, with the median values marked on the graph and listed at the top. Statistical significance (Mann-Whitney test, two-sided) and a schematic representation of the DNA fiber combing assay conditions are also presented. **g**. Western blots showing DNA2 and WRN depletion upon siRNA-mediated knockdown.

Supplementary Figure 4

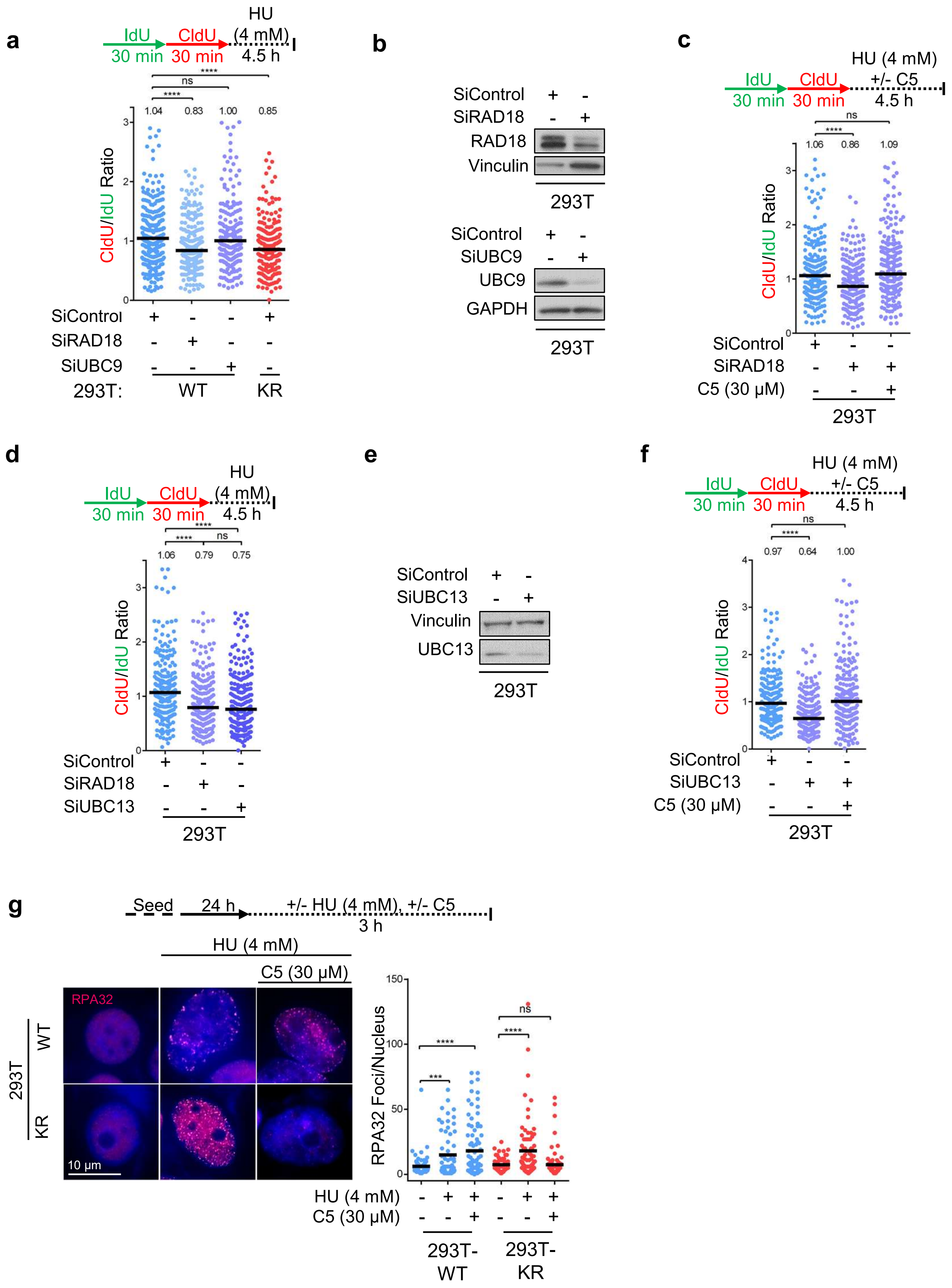

Supplementary Fig. 4. Impact of PCNA modification by ubiquitin or SUMO on fork stability. a. Depletion of RAD18, but not of UBC9, results in HU-induced nascent strand degradation. The ratio of CldU to IdU tract lengths is presented, with the median values marked on the graph and listed at the top. Asterisks indicate statistical significance (Mann-Whitney test, two-sided). A schematic representation of the DNA fiber combing assay conditions is also presented. **b**. Western blots showing RAD18 and UBC9 depletion upon siRNA-mediated knockdown. **c**. Depletion of RAD18 results in HU-induced nascent strand degradation mediated by DNA2. The ratio of CldU to IdU tract lengths is presented, with the median values marked on the graph and listed at the top. Asterisks indicate statistical significance (Mann-Whitney test, two-sided). A schematic representation of the DNA fiber combing assay conditions is also presented. **d.** Depletion of UBC13 results in HU-induced nascent strand degradation, similar to depletion of RAD18. The ratio of CldU to IdU tract lengths is presented, with the median values marked on the graph and listed at the top. Asterisks indicate statistical significance (Mann-Whitney test, two-sided). A schematic representation of the DNA fiber combing assay conditions is also presented. **e**. Western blot showing UBC13 depletion upon siRNA-mediated knockdown. **f.** Depletion of UBC13 results in HU-induced nascent strand degradation mediated by DNA2. The ratio of CldU to IdU tract lengths is presented, with the median values marked on the graph and listed at the top. Asterisks indicate statistical significance (Mann-Whitney test, two-sided). A schematic representation of the DNA fiber combing assay conditions is also presented. **g.** Immunofluorescence experiment showing that HU-induced RPA32 foci formation is suppressed by DNA2 inhibition in 293T-K164R cells, but not in wildtype cells. At least 75 cells were quantified for each condition. The mean values are marked on the graph, and asterisks indicate statistical significance (t-test, two-tailed, unequal variance). Representative micrographs are also shown. Source data are provided as a Source Data file.

Supplementary Figure 5

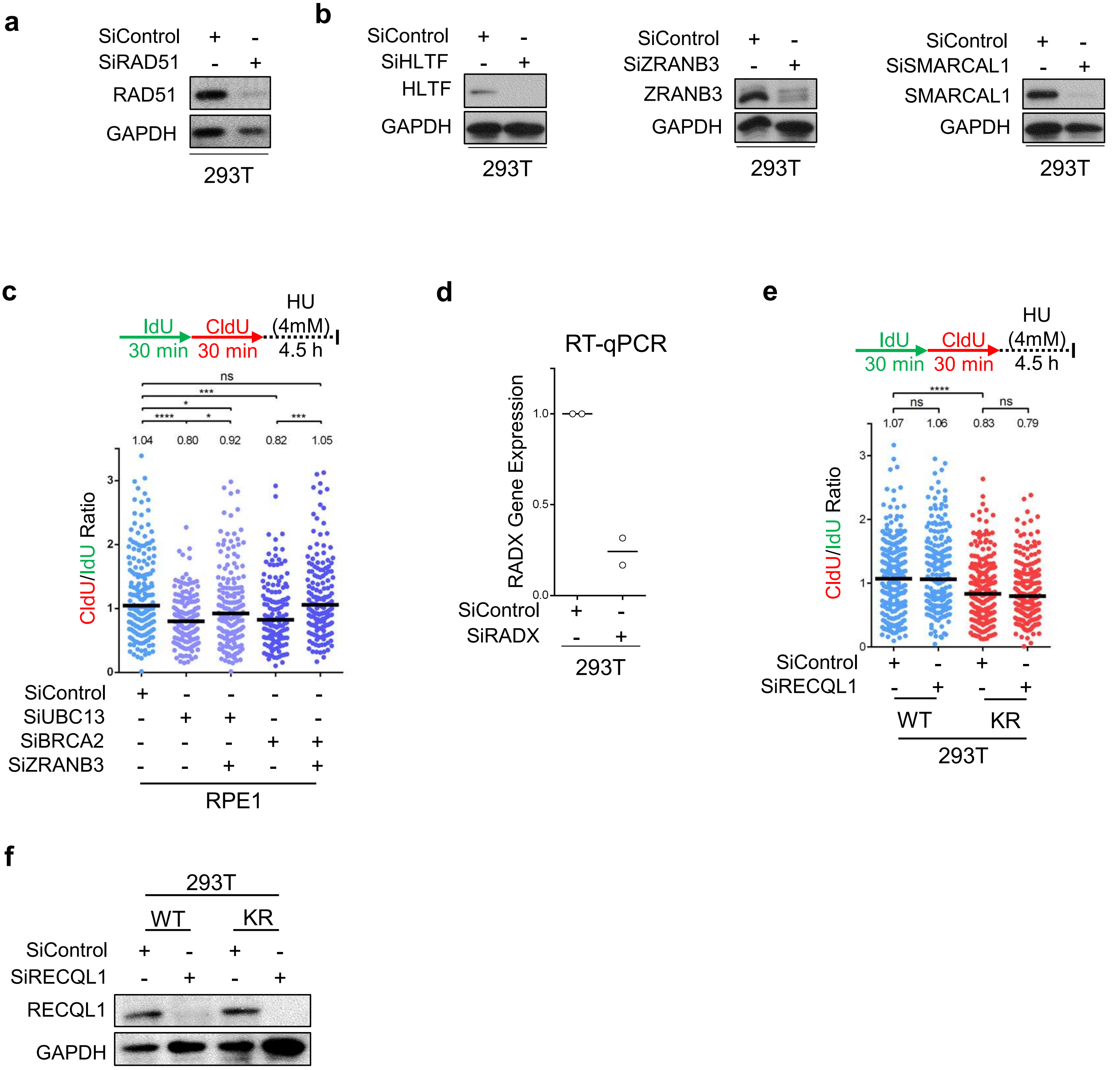

Supplementary Fig. 5. Impact of fork reversal on nascent strand degradation in PCNA-K164R cells. a. Western blot confirming RAD51 depletion upon siRNA-mediated knockdown. **b.** Western blots showing depletion of HLTF, ZRANB3 and SMARCAL1 upon siRNA-mediated knockdown. **c.** Knockdown of ZRANB3 fully suppresses HU-induced nascent tract degradation in BRCA2-depleted RPE1 cells, but only partially suppresses HU-induced nascent tract degradation in UBC13-depleted cells. The ratio of CldU to IdU tract lengths is presented, with the median values marked on the graph and listed at the top. Asterisks indicate statistical significance (Mann-Whitney test, two-sided). A schematic representation of the assay conditions is also presented. **d**. RT-qPCR experiment showing reduction in RADX mRNA levels upon siRNA-mediated knockdown. Two technical replicates are shown, with the lines representing the means. (No antibody was available to us to verify the depletion by western blot.) **e.** RECQL1 is not involved in the nascent tract degradation observed in K164R cells upon HU exposure, as its knockdown does not induce fork degradation in wildtype cells, and does not the affect the degradation observed in KR cells. The ratio of CldU to IdU tract lengths is presented, with the median values marked on the graph and listed at the top. Asterisks indicate statistical significance (Mann-Whitney test, two-sided). A schematic representation of the assay conditions is also presented. **f.** Western blot confirming RECQL1 depletion upon siRNA-mediated knockdown. Source data are provided as a Source Data file.

Supplementary Figure 6

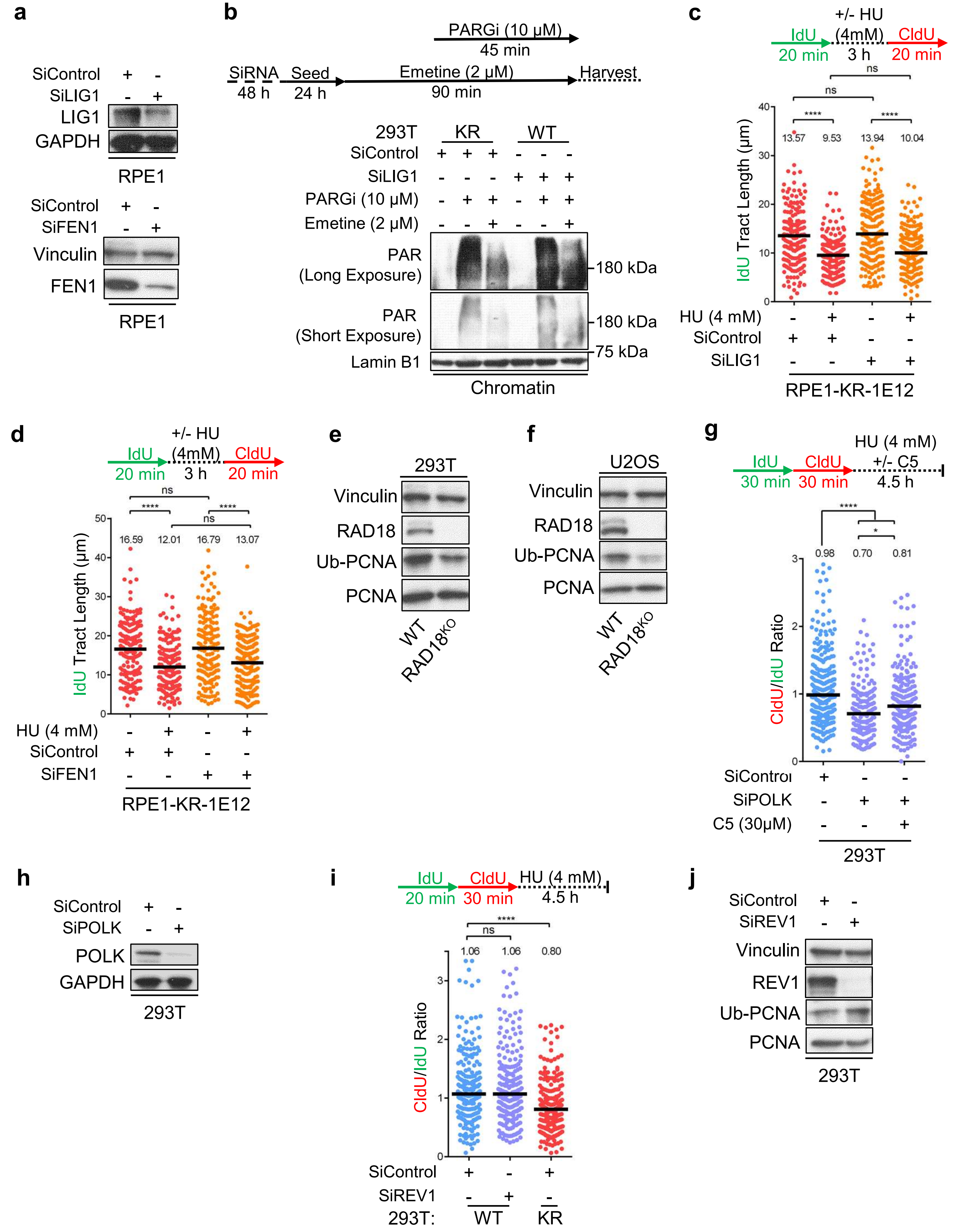

Supplementary Fig. 6. Impact of Okazaki fragment maturation factors and TLS polymerases on replication fork protection a. Western blots confirming LIG1 and FEN1 depletion upon siRNA-mediated knockdown. **b.** Chromatin fractionation experiment showing that PAR chain formation in PCNA-K164R and LIG1-depleted 293T cells is suppressed upon incubation with 2µM emetine for 90 min. Cells were treated as indicated with a PARG inhibitor (PARGi) for 45min prior to harvesting to block PAR chain removal. Chromatin-associated LaminB1 was used as loading control. **c, d**. DNA fiber combing assay showing that LIG1 (**c**) or FEN1 (**d**) depletion in RPE1 cells does not further increase fork progression rate and HU-induced degradation in K164R cells. The quantification of the IdU tract length is presented, with the median values marked on the graph and listed at the top. Asterisks indicate statistical significance (Mann-Whitney test, two-sided). A schematic representation of the assay conditions is also presented. **e, f.** Western blots showing the loss of RAD18 expression in 293T (**e**) and U2OS (**f**) RAD18-knockout cells, as well as the level of PCNA ubiquitination in these cells. **g**. POLK depletion results in HU-induced nascent strand degradation which is partially dependent on DNA2 enzymatic activity. The ratio of CldU to IdU tract lengths is presented, with the median values marked on the graph and listed at the top. Asterisks indicate statistical significance (Mann-Whitney test, two-sided). A schematic representation of the fiber combing assay conditions is also presented. **h**. Western blot confirming POLK depletion upon siRNA-mediated knockdown. **i**. DNA fiber combing assay showing that REV1 depletion does not cause degradation of arrested replication forks. The ratio of CldU to IdU tract lengths is presented, with the median values marked on the graph and listed at the top. Asterisks indicate statistical significance (Mann-Whitney test, two-sided). A schematic representation of the assay conditions is also presented. **j**. Western blot confirming REV1 depletion upon siRNA-mediated knockdown. Source data are provided as a Source Data file.

Supplementary Figure 7

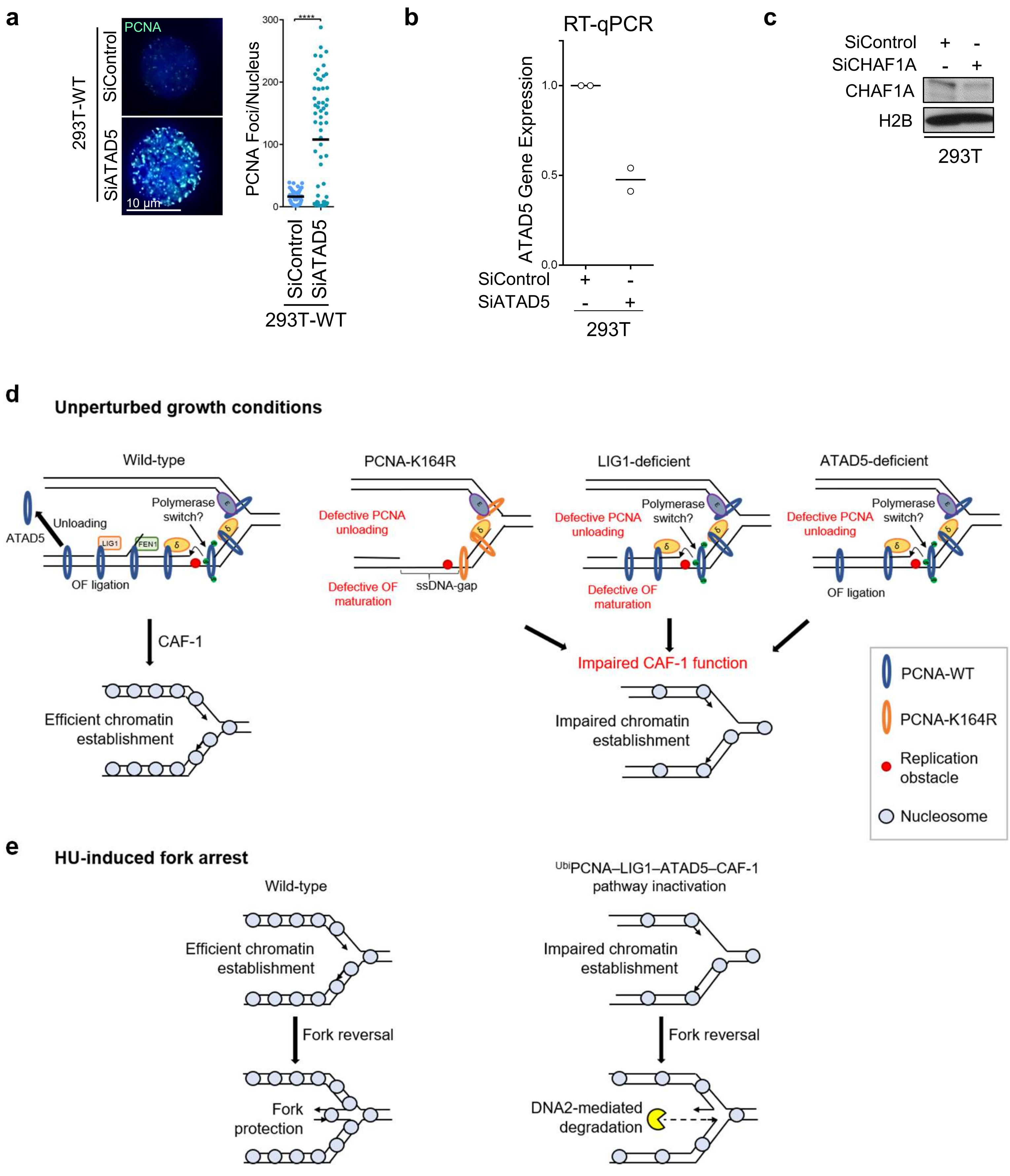

Supplementary Fig. 7. PCNA chromatin retention in ATAD5-depleted cells. A. Immunofluorescence experiment showing increased PCNA chromatin retention upon ATAD5 depletion. At least 65 cells were quantified for each condition. The mean values are marked on the graph, and asterisks indicate statistical significance (t-test, two-tailed, unequal variance). Representative micrographs are shown. **b**. RT-qPCR experiment showing reduction in ATAD5 mRNA levels upon siRNA-mediated knockdown. Two technical replicates are shown, with the lines representing the means. **c**. Western blot confirming CHAF1A depletion upon siRNA-mediated knockdown. **d, e**. Proposed model depicting the ^{Ubi}PCNA–LIG1–ATAD5–CAF-1 genetic pathway. Under unperturbed growth conditions (**d**), PCNA ubiquitination is required for efficient lagging strand synthesis by mediating gap-filling behind progressing replication forks. Forks arrested at endogenous lesions, whether on the lagging or leading strand, require PCNA ubiquitination-mediated TLS for restart. On the leading strand (not shown), repriming downstream of the lesion (perhaps by PrimPol) mediates replication restart in the absence of PCNA ubiquitination. In contrast, on the lagging strand, continuous initiation of new Okazaki fragments by Polα allows unhindered movement of the replication fork, alleviating the need for downstream repriming. In the absence of PCNA ubiquitination, persistent gaps are formed between the lesion and the previous OF. These gaps interfere with OF maturation and subsequent PCNA unloading. By sequestering the CAF-1 chromatin assembly complex, PCNA retention on the lagging strand alters the efficiency of chromatin establishment which results in replication forks encountering a sparse chromatin organization. As nucleosomal spacing regulates OF priming, inactivation of the ^{Ubi}PCNA–LIG1–ATAD5–CAF-1 pathway results in longer OFs which initiate further ahead of lagging strand synthesis. HU treatment (**e**) induces fork arrest and reversal. In PCNA-K164R, cells fork reversal is reduced but forks that do reverse, through the activity of SMARCAL1 and potentially ZRANB3, have an asymmetric structure with a 5'-overhang in the regressed arm because of the increased length of the OF

fragment previously generated. This abnormal reversed fork structure is a preferred substrate for DNA2, leading to uncontrolled resection. Source data are provided as a Source Data file.

Supplementary Figure 8

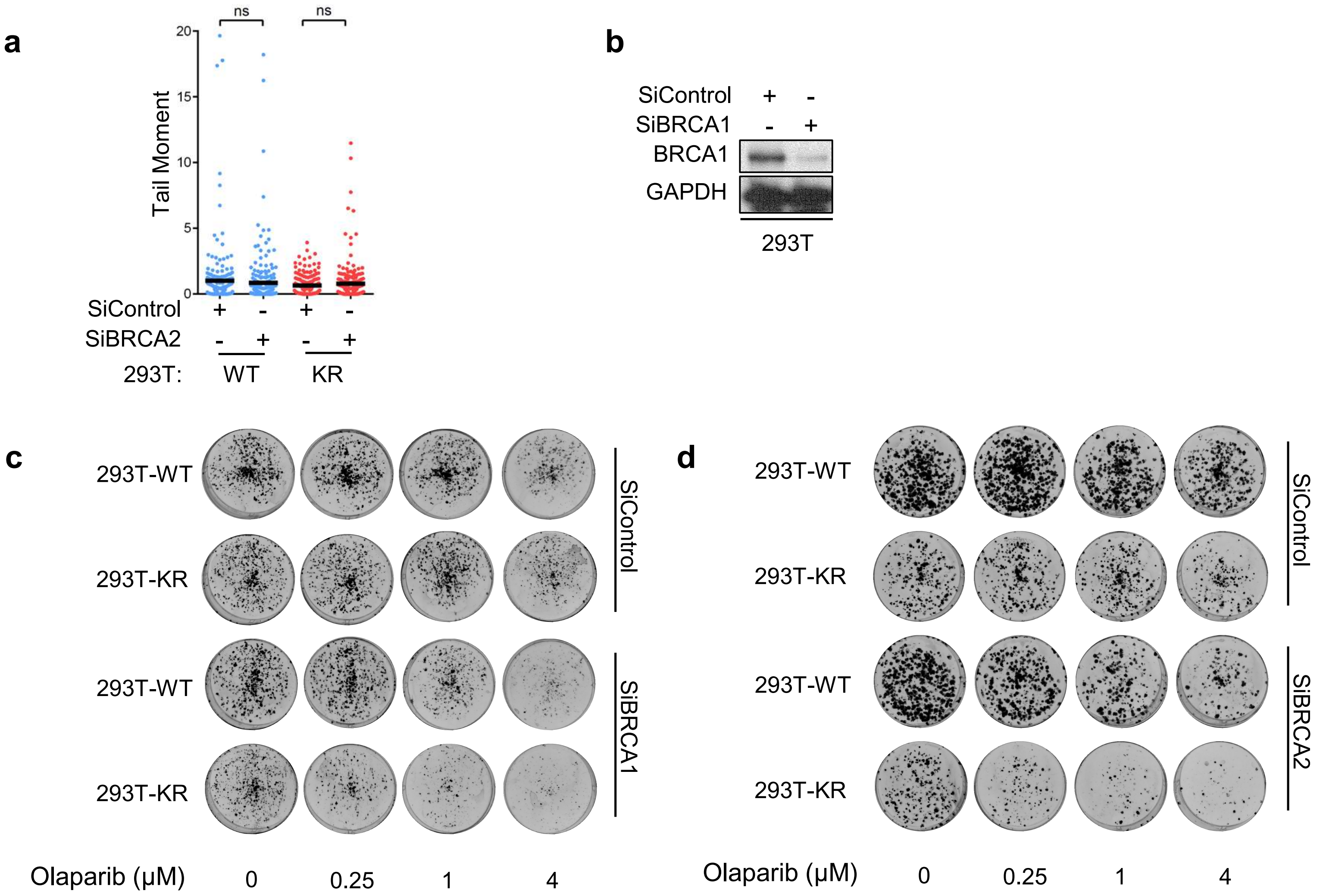

Supplementary Fig. 8. Genetic interaction between PCNA ubiquitination and the BRCA pathways. a. Neutral comet assay showing no increase in DSBs upon BRCA2 knockdown in 293T-K164R cells under normal growth conditions. At least 100 cells were quantified for each condition. The mean values are marked on the graph, and asterisks indicate statistical significance (t-test, two-tailed, unequal variance). **b**. Western blot showing BRCA1 depletion upon siRNA-mediated knockdown. **c, d**. Representative images of the clonogenic assays showing increased olaparib sensitivity of BRCA1-depleted (**c**) or BRCA2-depleted (**d**) 293T-K164R cells. Source data are provided as a Source Data file.
