# Supplementary material for: Ubiquitinated-PCNA protects replication forks from DNA2-mediated degradation by regulating Okazaki fragment maturation and chromatin assembly
Source: Nat Commun. 2020 May 1;11:2147. doi: 10.1038/s41467-020-16096-w (PMC7195461; doi:10.1038/s41467-020-16096-w)
Supplement: Supplementary file 3 — Reporting Summary [file 41467_2020_16096_MOESM3_ESM.pdf]

nature research

Corresponding author(s): George-Lucian Moldovan

Last updated by author(s): Mar 29, 2020

Reporting Summary

Nature Research wishes to improve the reproducibility of the work that we publish. This form provides structure for consistency and transparency in reporting. For further information on Nature Research policies, see Authors & Referees and the Editorial Policy Checklist.

Statistics

For all statistical analyses, confirm that the following items are present in the figure legend, table legend, main text, or Methods section.

n/a	Confirmed	
☐	☒	The exact sample size (*n*) for each experimental group/condition, given as a discrete number and unit of measurement
☐	☒	A statement on whether measurements were taken from distinct samples or whether the same sample was measured repeatedly
☐	☒	The statistical test(s) used AND whether they are one- or two-sided<br>*Only common tests should be described solely by name; describe more complex techniques in the Methods section.*
☒	☐	A description of all covariates tested
☒	☐	A description of any assumptions or corrections, such as tests of normality and adjustment for multiple comparisons
☐	☒	A full description of the statistical parameters including central tendency (e.g. means) or other basic estimates (e.g. regression coefficient) AND variation (e.g. standard deviation) or associated estimates of uncertainty (e.g. confidence intervals)
☒	☐	For null hypothesis testing, the test statistic (e.g. *F*, *t*, *r*) with confidence intervals, effect sizes, degrees of freedom and *P* value noted<br>*Give P values as exact values whenever suitable.*
☒	☐	For Bayesian analysis, information on the choice of priors and Markov chain Monte Carlo settings
☒	☐	For hierarchical and complex designs, identification of the appropriate level for tests and full reporting of outcomes
☒	☐	Estimates of effect sizes (e.g. Cohen's *d*, Pearson's *r*), indicating how they were calculated

Our web collection on statistics for biologists contains articles on many of the points above.

Software and code

Policy information about availability of computer code

Data collection	1) DNA Fiber Combing: Leica SP5 Confocal Microscope.<br>2) Immunofluorescence: DeltaVision Elite Confocal Microscope.<br>3) Flow Cytometry: BD FACSCanto.<br>4) Gel Imager for MNase: Bio-Rad ChemiDoc MP Imaging System.
Data analysis	1) DNA Fiber Combing: LASX 3.3.0.16799<br>2) Immunofluorescence: ImageJ 1.52p<br>3) Flow Cytometry: BD FACSDiva 8.0.1<br>4) MNase Intensity Measurement: Icy 2.0.2.0<br>5) Comet Assay analyses: CometScore 2.0<br>6) Statistics and Data Figure Panels: GraphPad Prism 6<br>7) Primary Data recording: Microsoft Excel 16<br>8) Figure Panel Organization: Microsoft Powerpoint 16

For manuscripts utilizing custom algorithms or software that are central to the research but not yet described in published literature, software must be made available to editors/reviewers. We strongly encourage code deposition in a community repository (e.g. GitHub). See the Nature Research guidelines for submitting code & software for further information.

Data

Policy information about availability of data

All manuscripts must include a data availability statement. This statement should provide the following information, where applicable:

- Accession codes, unique identifiers, or web links for publicly available datasets
- A list of figures that have associated raw data
- A description of any restrictions on data availability

Data supporting the findings of this manuscript are available from the corresponding author upon reasonable request. The source data underlying Figs. 1a-h, 2a-f, 3a-c, 4a-e, 5a-d, 6a-g, and Supplementary Figs. 1a-e, 2a-s, 3a-f, 4a-j, 4a-c, 6a-b are provided as a Source Data file.

Field-specific reporting

Please select the one below that is the best fit for your research. If you are not sure, read the appropriate sections before making your selection.

☒ Life sciences ☐ Behavioural & social sciences ☐ Ecological, evolutionary & environmental sciences

For a reference copy of the document with all sections, see nature.com/documents/nr-reporting-summary-flat.pdf

Life sciences study design

All studies must disclose on these points even when the disclosure is negative.

Sample size	No statistical method was used to predetermine sample size. Sample size was determined based on previous experiments and relevant literature in the field (eg Taglialatela et al, PMID: 29053959; Lemacon et al, PMID: 29038425). Statistical significance was obtained with these sample size.
Data exclusions	For drug sensitivity assays, no data was excluded from the analysis. For DNA fiber and immunofluorescence data, where appropriate, outlier tests were performed in order to obtain the best representative central tendency. As suitable, either Robust regression and Outlier removal (GraphPad Prism) or Grubb's test were used.
Replication	Western blot experiments were reproduced at least three times. DNA fiber assays, immunofluorescence and comet assays were performed at least twice. All results were reproducible.
Randomization	Samples were analyzed in random order. Other than that, randomization is not applicable to the assays performed here.
Blinding	For DNA fiber assays, samples were tested at least once under blinding conditions, and results were reproduced. Blinding is less feasible for the other assays (such as those western blot-based) and was not performed. However, these results were reproduced independently by at least two different authors.

Reporting for specific materials, systems and methods

We require information from authors about some types of materials, experimental systems and methods used in many studies. Here, indicate whether each material, system or method listed is relevant to your study. If you are not sure if a list item applies to your research, read the appropriate section before selecting a response.

Materials & experimental systems

n/a	Involved in the study	
☐	☒	Antibodies
☐	☒	Eukaryotic cell lines
☒	☐	Palaeontology
☒	☐	Animals and other organisms
☒	☐	Human research participants
☒	☐	Clinical data

Methods

n/a	Involved in the study	
☒	☐	ChIP-seq
☒	☐	Flow cytometry
☒	☐	MRI-based neuroimaging

Antibodies

Antibodies used	PCNA (Cell Signaling Technology 2586); Ubiquityl-PCNA Lys164 (Cell Signaling Technology 13439); CHK2 (Cell Signaling Technology 2662); pCHK2-T68 (Cell Signaling Technology 2661); CHK1 (Cell Signaling Technology 2360); pCHK1-S317 (Cell Signaling Technology 2344); BRCA2 (Bethyl A303-434A); EXO1 (Santa Cruz Biotechnology sc-56092); WRN (Santa Cruz Biotechnology sc-5629); CTIP (Santa Cruz Biotechnology sc-271339); MUS81 (Santa Cruz Biotechnology sc-47692); RAD18 (Cell Signaling Technology 9040); UBC9 (Santa Cruz Biotechnology sc-10759); RAD51 (Santa Cruz Biotechnology sc-8349); ZRANB3 (Bethyl A303-033A); HLTF (Santa Cruz Biotechnology sc-398357); SMARCAL1 (Santa Cruz Biotechnology sc-376377); RECQL1 (Santa Cruz Biotechnology sc-166388); PAR chains (ENZO ALX-804-220); LaminB1 (Abcam ab16048); LIG1 (Santa Cruz

	Biotechnology sc-271678); REV1 (Santa Cruz Biotechnology sc-393022); POLK (Santa Cruz Biotechnology sc-166667); CHAF1A (Cell Signaling Technology 5480); BRCA1 (Santa Cruz Biotechnology sc-642); Vinculin (Santa Cruz Biotechnology sc-73614); GAPDH (Santa Cruz Biotechnology sc-47724); FEN1 (Santa Cruz Biotechnology sc-28355); UBC13 (Santa Cruz Biotechnology sc-376470).
Validation	Antibodies were validated by western blots using siRNA-mediated knockdown. The data is presented in the manuscript.

Eukaryotic cell lines

Policy information about cell lines

Cell line source(s)	293T, RPE1, HeLa, and U2OS cells were obtained from ATCC
Authentication	Authentication was performed regularly based on morphology and gene/protein expression (in case of genetic alterations)
Mycoplasma contamination	Cell lines were tested for mycoplasma contamination, and if found contaminated, were treated with plasmocin
Commonly misidentified lines (See ICLAC register)	None of the cell lines used are listed on the ICLAC register version 10
